# Ultrastructure of isolated mouse ovarian follicles cultured *in vitro*

**DOI:** 10.1186/1477-7827-9-3

**Published:** 2011-01-13

**Authors:** Stefania A Nottola, Sandra Cecconi, Serena Bianchi, Cecilia Motta, Gianna Rossi, Maria A Continenza, Guido Macchiarelli

**Affiliations:** 1 Department of Anatomy, Histology, Forensic Medicine and Orthopaedics, "La Sapienza" University of Rome, Rome, Italy; 2 Department of Health Sciences, University of L'Aquila, L'Aquila, Italy; 3 Centre of Microscopy, University of L'Aquila, L'Aquila, Italy

## Abstract

**Background:**

*In vitro *maturation of ovarian follicles, in combination with cryopreservation, might be a valuable method for preserving and/or restoring fertility in mammals with impaired reproductive function. Several culture systems capable of sustaining mammalian follicle growth *in vitro *have been developed and many studies exist on factors influencing the development of *in vitro *grown oocytes. However, a very few reports concern the ultrastructural morphology of *in vitro *grown follicles.

**Methods:**

The present study was designed to evaluate, by transmission and scanning electron microscopy, the ultrastructural features of isolated mouse preantral follicles cultured *in vitro *for 6 days in a standard medium containing fetal calf serum (FCS). The culture was supplemented or not with FSH.

**Results:**

The follicles cultured in FCS alone, without FSH supplementation (FCS follicles), did not form the antral cavity. They displayed low differentiation (juxta-nuclear aggregates of organelles in the ooplasm, a variable amount of microvilli on the oolemma, numerous granulosa cell-oolemma contacts, signs of degeneration in granulosa cell compartment). Eighty (80)% of FSH-treated follicles formed the antral cavity (FSH antral follicles). These follicles showed various ultrastructural markers of maturity (spreading of organelles in ooplasm, abundant microvilli on the oolemma, scarce granulosa cell-oolemma contacts, granulosa cell proliferation). Areas of detachment of the innermost granulosa cell layer from the oocyte were also found, along with a diffuse granulosa cell loosening compatible with the antral formation. Theca cells showed an immature morphology for the stage reached. Twenty (20)% of FSH-treated follicles did not develop the antral cavity (FSH non-antral follicles) and displayed morphological differentiation features intermediate between those shown by FCS and FSH antral follicles (spreading of organelles in the ooplasm, variable amount of microvilli, scattered granulosa cell-oolemma contacts, signs of degeneration in granulosa cell compartment).

**Conclusions:**

It is concluded that FSH supports the *in vitro *growth of follicles, but the presence of a diffuse structural granulosa cell-oocyte uncoupling and the absence of theca development unveil the incomplete efficiency of the system. The present study contributes to explain, from a morphological point of view, the effects of culture conditions on the development of mouse *in vitro *grown follicles and to highlight the necessity of maintaining efficient intercellular communications to obtain large numbers of fully-grown mature germ cells.

## Background

*In vitro *culture and maturation of preantral ovarian follicles currently represents one of the most important tools of investigation in the field of assisted reproduction. This technique, in combination with cryopreservation, might be a valuable method for preserving and/or restoring fertility in mammals with impaired reproductive function, ultimately achieving *in vitro *growth of viable oocytes competent for fertilization [1]. The low temperature has been demonstrated useful to store gametes in several mammalian species such as mouse [2-4], sheep [5,6] and human [for review, see: 7]. After thawing, one strategy for yielding mature oocytes may be to isolate small (preantral) follicles (that result tolerant to cryodamage) [1] from the cryopreserved ovarian tissue and to subject them to subsequent *in vitro *culture [2,8-16].

In recent years, numerous *in vitro *culture systems for ovarian mammalian follicles and immature oocytes have been designed to study regulative processes occurring during folliculogenesis and oogenesis. These studies contributed to the development of new reproductive biotechnologies, including clinical application in the treatment of human infertility [1,15,17-22]. At present, follicles at various stages of development collected from both fresh or cryopreserved ovarian tissue have the potentiality to grow *in vitro*. However, culture procedures have not been yet optimized. This is partially due to the heterogeneity of the follicle population present in the adult ovarian tissue and to the extreme variability of culture systems and media, including considerable variations in length of the culture period in relation to different animal species [1,18,23].

Fertilizable oocytes and live offspring following embryo transfer were firstly obtained from mouse preantral follicles, cultured either intact [24] or devoid of theca cell (TC) layers (oocyte-granulosa cell - GC-complexes) [25-27]. Then, many papers have been published on the culture of small follicles from mouse [1,28] or large mammals such as pig [29-31], sheep [32], bovine [17,29,33,34], primates [17] and humans [14,20,35,36]. These studies pointed out that mouse is the most handling source for experimental *in vitro *model. In fact, mouse oocytes can acquire full developmental competence under very different culture conditions [24,25,37-42]. Thus, setting up an *in vitro *grown (IVG) mouse follicle model for the morphological studies is fundamental for the validation of IVG technologies and may provide a useful background for the studies in large mammals [43]. However, up to now only a few and not comprehensive microscopical studies concerned with IVG mouse follicles.

During the last decades, electron microscopy provided significant data on the morphological changes of mammalian ovarian follicle development *in vivo *[44-49]. More recently, several studies reported a few ultrastructural data on isolated preantral ovarian follicles of various mammals subjected *in vitro *to different culture conditions. These observations focused on early maturation of human primordial follicles [14], changes of mouse TCs [50] and rat GC-oolemma contacts [51] or degenerative aspects of bovine follicles [52] in culture. The ultrastructure of preantral mouse follicle after cryopreservation was studied in mouse [2,16]. Other studies concerned follicle ultrastructure in human [53,54] and goat [55,56] organ cultures. These papers agree with the fact that transmission electron microscopy (TEM) is an irreplaceable tool for the fine study of the ovarian follicle morphodynamics. However, a comprehensive and systematic ultrastructural survey of IVG isolated preantral follicles grown *in vitro *in basic conditions is lacking in the literature. In addition, to our knowledge, the fine surface morphology of IVG follicles as revealed by scanning electron microscopy (SEM) has been previously addressed only in a preliminary report of our group [57].

Aims of the present study were: 1. to analyze the ultrastructural features of IVG mouse preantral follicles cultured with TC layer, evaluating follicle morphology by a combined TEM/SEM ultrastructural approach, to describe fine intracellular structures and three-dimensional details; 2. to compare the ultrastructural features of the follicles cultured in presence or absence of FSH supplementation.

## Methods

### Preantral follicle isolation and culture

Five consecutive experiments were performed. All the experiments were carried out in accordance with the procedure described in the guidelines for the care and use of laboratory animals approved by the Animal Care Committee of the University of L'Aquila. A total of 20 Swiss CD1 female mice (Harlan, Udine, Italy), aged 24-26 days, killed by cervical dislocation, were used in these experiments. Preantral follicles (N = 500) were mechanically isolated from the ovaries (together with a small clump of thecal-stromal tissue attached) under a stereo-microscope using fine needles and collected with a micropipette. Only those showing a centrally located oocyte within an intact basement membrane, with no apparent sign of necrosis were selected for further culture (N = 350). To this end follicles were individually cultured in 25 μl of culture medium in 96-V-well microtitre plates (Greiner Labortechnik, LTD), overlaid with 70 ml of mineral oil (embryo tested, d = 0.84 g/ml). Culture medium was Alpha Minimal Essential Medium (α-MEM) supplemented with 1% ITS (insulin, 5 μg/ml; transferrin, 5 μg/ml; and sodium selenite, 5 ng/ml), antibiotics (penicillin, 100 U/ml; streptomycin, 100 mg/ml), and 5% fetal calf serum (FCS) supplemented or not with highly purified ovine FSH (100 mIU/ml, National Institute of Diabetes and Digestive and Kidney Diseases (NIDDK)-o-FSH-19-SIAFP, BIO). All the other chemicals used in culture were purchased from Sigma Chemical Company (St. Louis, MO, USA). Both follicles cultured in FCS alone, without FSH supplementation (FCS follicles, N = 150) and FSH-treated follicles (N = 200) were incubated at 37°C in 5% CO_2 _in air and saturated humidity for 6 days. Culture medium was changed every other day.

### Electron microscopy

At the end of the culture period, follicles from both groups (N = 60) were destined to electron microscopy analysis.

Thirty (30) follicles were fixed in 1.5% glutaraldehyde in 0.1 M PBS and then processed for light microscopy (LM) and TEM according to the procedures described by Motta et al. [46]. Semi-thin sections, cut with a glass knife and mounted on glass, were stained with methylene blue and observed by LM. Ultra-thin sections cut with a diamond knife and mounted onto copper grids, were double stained with uranyl acetate and lead citrate. TEM observations were performed by means of Zeiss EM 10 and Philips TEM CM100 Electron Microscopes.

The remaining 30 specimens were fixed in 2.5% glutaraldehyde in 0.1 M PBS and processed for conventional SEM or for digestion of the extracellular matrix [45,58]. By this method, the cell framework of follicles was preserved whereas extracellular tissue components, including collagen fibrils, were removed. Samples were observed in Philips XL-30-CP and Hitachi S-4000 scanning electron microscopes.

### Statistical analysis

Follicular growth was evaluated by phase contrast microscopy (PCM) measuring follicular and oocyte diameters (in μm) from day 1 to day 6 of culture.

For the image analysis study, sections were made at the midpoint of the follicles, in order to minimize bias due to tangential sectioning. The presence and amount of the transzonal processes (TZPs) of the innermost layer of the GCs surrounding the oocytes of FCS follicles and FSH-treated follicles was evaluated by TEM on sections made at several planes (magnification: x6300). Images were further enlarged on the PC screen, in order to easily recognize and count TZPs. Values were expressed in number of visible portions of TZPs per 20 μm^2 ^of zona pellucida area.

Statistical data were shown as mean ± SD. *P*-value and statistical significance were evaluated by Student's t test [59]. Significance was defined as *P *< 0.05.

## Results

### Follicle culture

At the end of a 6 day-culture period, all FCS follicles underwent a small increase in size (from 160 ± 10 μm to 200 ± 10 μm; *P *> 0.05) and did not develop the antral cavity (AC). By contrast, a high percentage (80%) of FSH-treated follicles increased significantly in diameter (from 160 ± 10 μm to 420 ± 20 μm; *P *< 0.05) and formed variable sized ACs. By PCM the AC appeared as a translucent area in the GC mass that could also comprise about half of the follicle. On the basis of the above observations, follicles were sorted into three groups: FCS follicles, FSH-treated follicles with a visible AC (FSH antral follicles), and FSH-treated follicles without a visible AC (FSH non-antral follicles). Growth was also observed in the germinal component, as oocyte diameter increased from 62 ± 1 μm up to 73 ± 1 μm (*P *< 0.01) without differences among the various groups [39,40].

After culture, 60 intact follicles (20 FCS follicles, 20 FSH antral follicles and 20 FSH non-antral follicles) were chosen and assigned to electron microscopy examination. Specifically, 10 follicles from each group were prepared for LM-TEM analysis whereas the remaining follicles were destined to SEM examination.

### General appearance of IVG follicles at day 6

**Figure 1:**
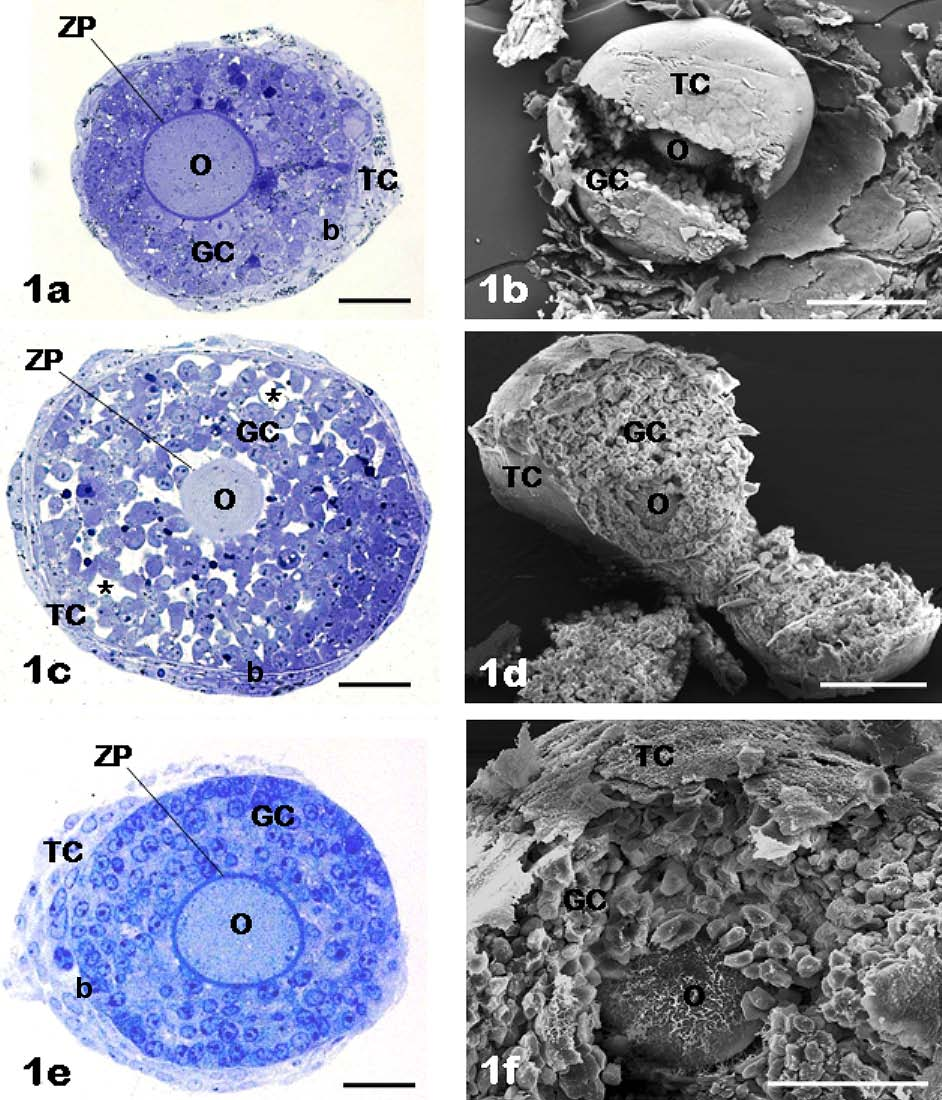
**General appearance of *in vitro *grown follicles**. FCS follicles (panels a, b); FSH antral follicles (panels c, d); FSH non-antral follicles (panels e, f). The general appearance of cultured follicles is shown by LM (panels a, c, e) and SEM (panels b, d, f). Panels a-f, O: oocyte; GC: granulosa cells; TC: theca cells. Panels a, c, e, ZP: zona pellucida; b: basement membrane. Panel c, asterisks: fluid-filled spaces. Bar is: 30 μm (panel a); 100 μm (panels b, d); 50 μm (panels c, f); 45 μm (panel e).

As seen by LM or SEM, all follicles maintained a round shape during culture. All follicular main structural elements were present (TCs, basement membrane, GCs, zona pellucida and oocyte) (Figure 1). All follicles showed round shaped and centrally located intact oocytes with a normally structured zona pellucida (Figure 1a, c, e). FCS follicles showed 4-5 layers of cuboidal GCs (Figure 1a, b); FSH-treated follicles showed more numerous GCs distributed in a higher number of layers (6-8) (Figure 1c, d, e, f). FSH antral follicles presented fluid-filled spaces among somatic cells (Figure 1c), while FCS and FSH non-antral follicles showed a compact granulosa layer (Figure 1a, b, e, f). All follicles showed an intact but thin, immature theca layer (Figure 1).

### Main ultrastructural features of IVG follicles at day 6

#### FCS follicles

**Figure 2:**
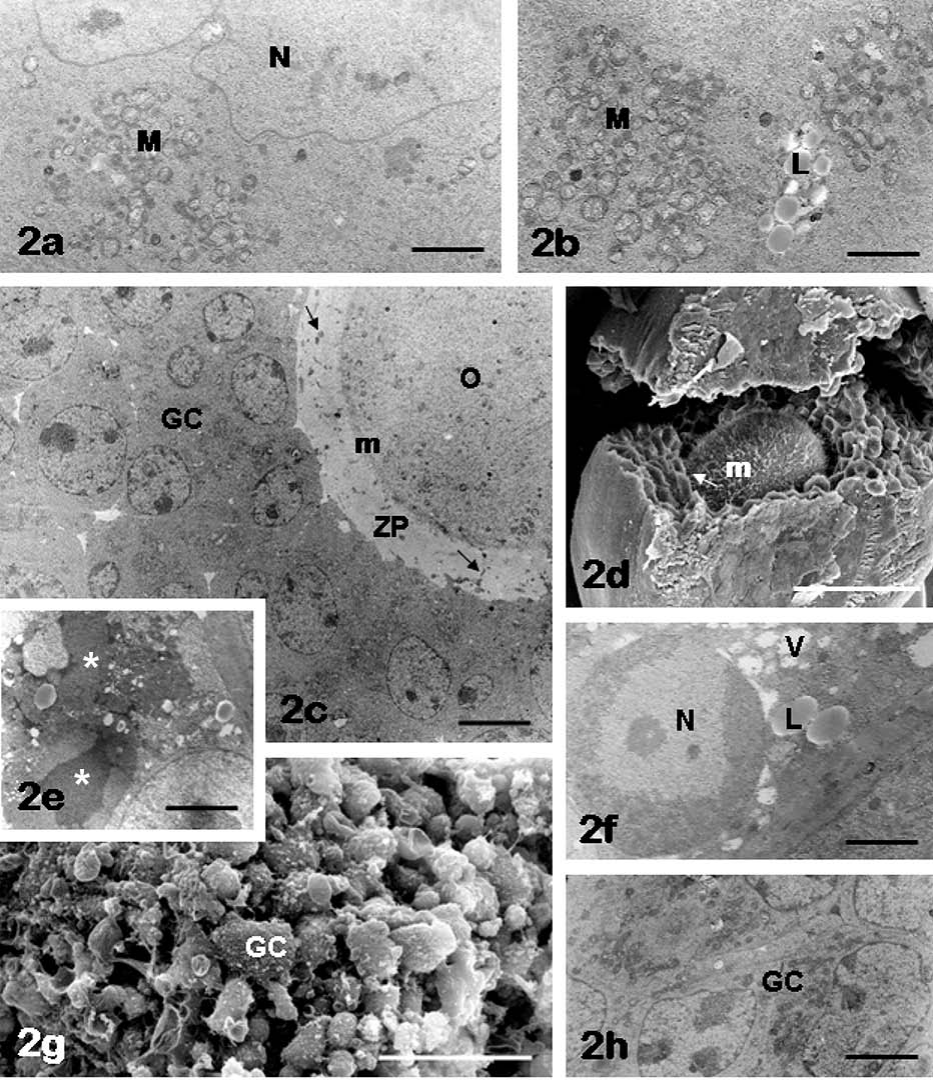
**FCS follicles: oocyte and granulosa cells**. Note the presence of juxta-nuclear aggregates of mitochondria (M) and lipid droplets (L) in the oocyte (panels a, b). Scarce (panel c) or more abundant (panel d) microvilli (m) are present on the oolemma. Damaged granulosa cells (asterisks, panel e) are seen around the oocyte, sometimes showing chromatin margination in the nucleus (N), vacuoles (V) and lipid droplets (L) in the cytoplasm (panel f). A diffuse blebbing on the granulosa cell (GC) surface is also observable in panel g. Granulosa cells (GC) with normal appearance are clearly recognizable in panels c, h. Panel a, N: oocyte nucleus; panels c, d, arrows: granulosa cell cytoplasmic projections crossing the zona pellucida (ZP) to reach the oocyte (O) surface (transzonal processes). Bar is: 2 μm (panel a); 1.8 μm (panel b); 5 μm (panel c); 52 μm (panel d); 2.8 μm (panel e); 1.5 μm (panel f); 40 μm (panel g); 2.1 μm (panel h). Panels a-c, e, f, h: TEM; panels d, g: SEM.

*Oocyte: *the nuclear envelope was continuous and irregular in shape because provided with folds and invaginations (Figure 2a). The ooplasm showed accumulation in the juxta-nuclear region of cytoplasmic organelles, especially aggregates of rounded mitochondria and lipid droplets (Figure 2a, b). The ooplasm also showed some vacuolization. The oolemma was continuous and bordered with microvilli, variable in length and amount. GCs were closely adhering to the zona pellucida (Figure 2c), which was always apparently normal and crossed by numerous TZPs originating from the innermost layer of the GCs and reaching the oolemma (GC-oolemma contacts) (Figure 2c, d). The mean number ± SD per 20 μm^2 ^of TZPs was 29.90 ± 7.08 (Table 1).

**Table 1:** Morphometric evaluation of the presence of transzonal processes (TZPs) in IVG mouse ovarian follicles cultured with or without FSH for 6 days

	*FCS*	*FSH ANTRAL*	*FSH NON-ANTRAL*
***N° of TZPs/20 μm***^***2***^	29.90 ± 7.08^a^	6.80 ± 6.20^b^	14.20 ± 8.05^c^

^a,b^*P *< 0.0001; ^a,c ^*P *= 0.0002; ^b,c ^*P *= 0.0334

Values are expressed as mean ± SD

*Granulosa layer: *GCs formed a compact layer around the oocyte (Figure 2c). In about 40% of samples abundant residual debris of damaged GCs were present (Figure 2e). In these samples a great number of GCs exhibited clear signs of cell damage such as: abnormal distribution of nuclear chromatin, e.g. margination and karyolysis (Figure 2f); presence of cytoplasmic large vacuoles and voluminous lipid droplets (Figure 2e, f); surface alterations mainly characterized by diffuse blebbing of GC membranes revealed by SEM (Figure 2g). The remaining samples (60%) presented a normal appearance of GCs, characterized by a nucleus containing dispersed chromatin and one or more nucleoli. In addition, numerous organelles, mainly represented by mitochondria, were seen in their cytoplasm (Figure 2c, h).

**Figure 3:**
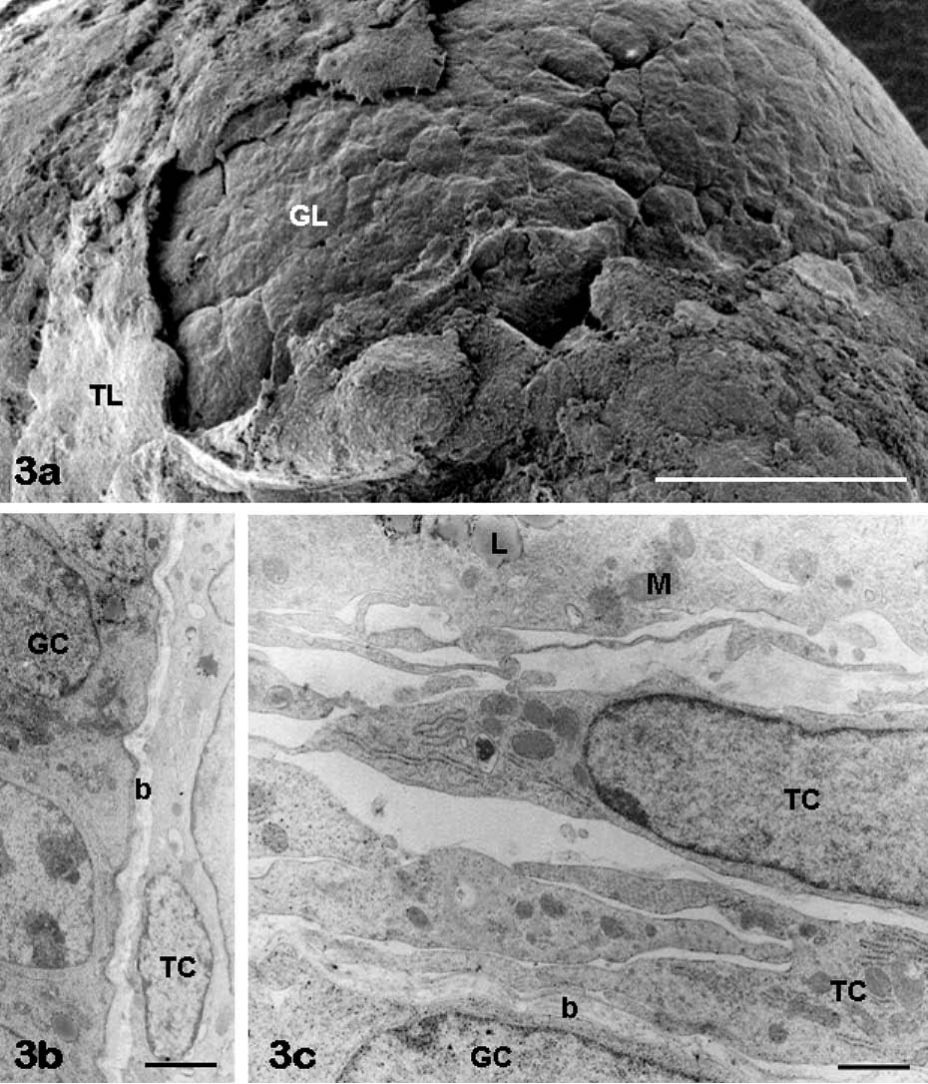
**FCS follicles: theca cells**. A thin theca cell layer (TL) is observable by SEM around the granulosa layer (GL) in panel a. Theca cells (TC) forming one or two-three layers are seen by TEM in panels b, c. Panels b, c, GC: granulosa cells; b: basement membrane. Panel c, M: theca cell mitochondria; L: theca cell lipid droplets. Bar is: 30 μm (panel a); 1 μm (panel b); 0.5 μm (panel c).

*Theca layer: *TCs usually distributed out of the healthy granulosa basement membrane in one (Figure 3a, b) or, more rarely, two (Figure 3c) continuous layers. TCs exhibited a large elongated nucleus with chromatin aggregates and a normal cytoplasm containing occasional lipid droplets and a few mitochondria (Figure 3b, c).

#### FSH antral follicles

**Figure 4:**
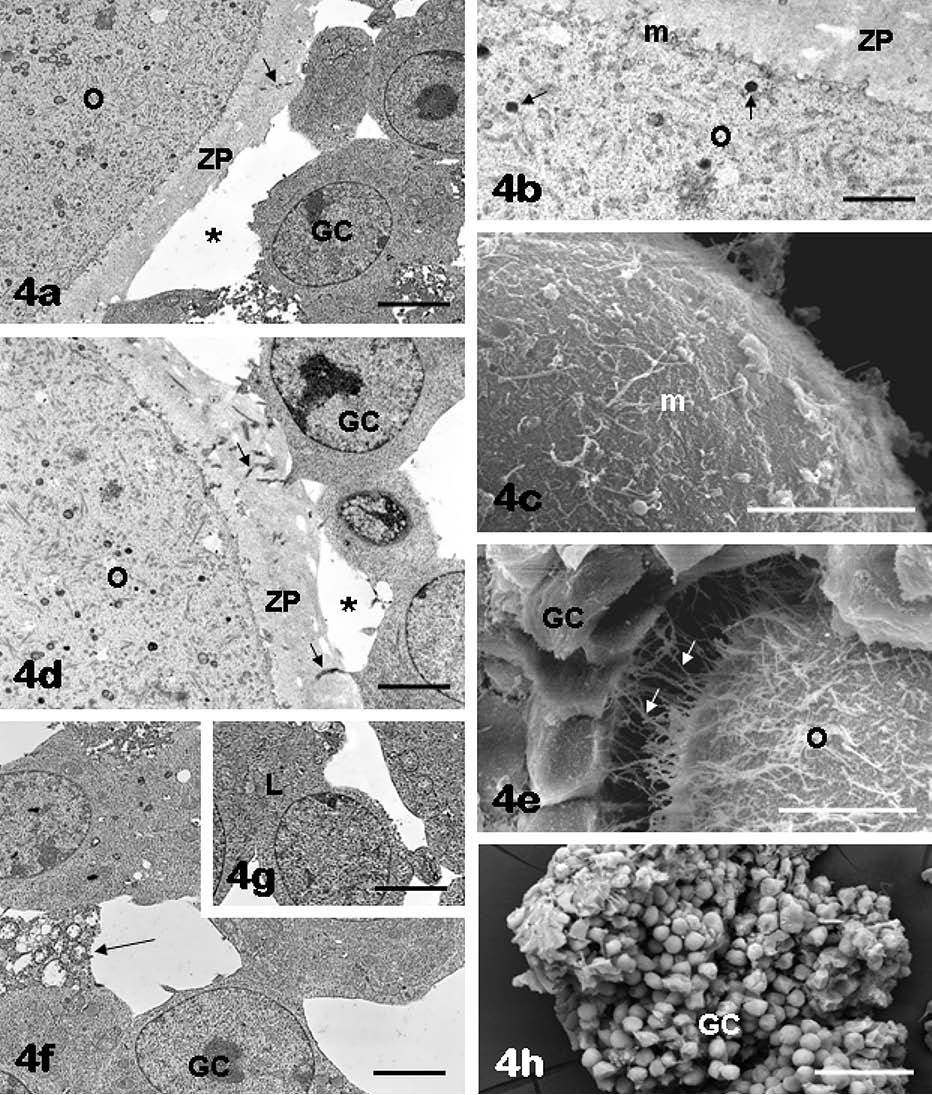
**FSH antral follicles: oocyte and granulosa cells**. The oocytes (O) contain numerous organelles, uniformly distributed in the ooplasm (panels a, b, d). Note the presence of scattered cortical granules (arrows) in the suboolemmal area (panel b). Numerous microvilli (m) can be observed on the oocyte surface (panels b, c). Cytoplasmic projections (arrows) stemming from the inner granulosa cell (GC) layer are seen crossing the zona pellucida (ZP) (panels a, d) and reaching the oocyte (transzonal processes) (panels d, e). Areas of detachment between inner granulosa cells and oocyte are also present (asterisks, panels a, d). Outer granulosa cells (GC) appeared irregularly rounded/polygonal and scarcely adherent each other (panels f, h). Uniformly dispersed chromatin and one or more nucleoli are seen in both inner (panels a, d) and outer (panels f, g) granulosa cells. Panel b, ZP: zona pellucida. Panel e, O: oocyte. Panel f, arrow: loss of contact among granulosa cells; panel g, L: lipid droplets in the granulosa cell cytoplasm. Bar is: 3.5 μm (panel a); 1 μm (panel b); 10 μm (panel c); 2 μm (panel d); 9 μm (panel e); 3 μm (panels f, g); 50 μm (panel h). Panels a, b, d, f, g: TEM; panels c, e, h: SEM.

*Oocyte: *the oocytes enclosed in FSH antral follicles showed an ooplasm provided with numerous scattered organelles mainly represented by rounded mitochondria with a few peripheral cristae, lipid droplets, primary and secondary lysosomes, aggregated or isolated vesicles of smooth endoplasmic reticulum and the fibrillar lattices typical of mouse oocytes (Figure 4a, b). The suboolemmal area contained sparse cortical granules and electron-dense particles along with membranes of smooth endoplasmic reticulum. The oolemma showed abundant and uniformly distributed microvilli protruding into the perivitelline space (Figure 4b, c). The innermost GCs showed areas of detachment from the oocyte, thus forming a discontinuous cell layer around a well preserved zona pellucida (Figure 4a, d). In those areas in which GCs were in close contact with the oocyte, long and tortuous TZPs were seen crossing the zona pellucida and reaching the oolemma (Figure 4a, d), where they intermingled with the microvilli covering the oocyte (Figure 4e). The GCs of the outer layer did not show these extensions. From a morphometric analysis the number of TZPs in FSH antral follicles (mean number ± SD per 20 μm^2 ^= 6.80 ± 6.20) appeared reduced in comparison with that found in FCS follicles, and this difference was highly significant (*P *< 0.0001) (Table 1).

**Figure 5:**
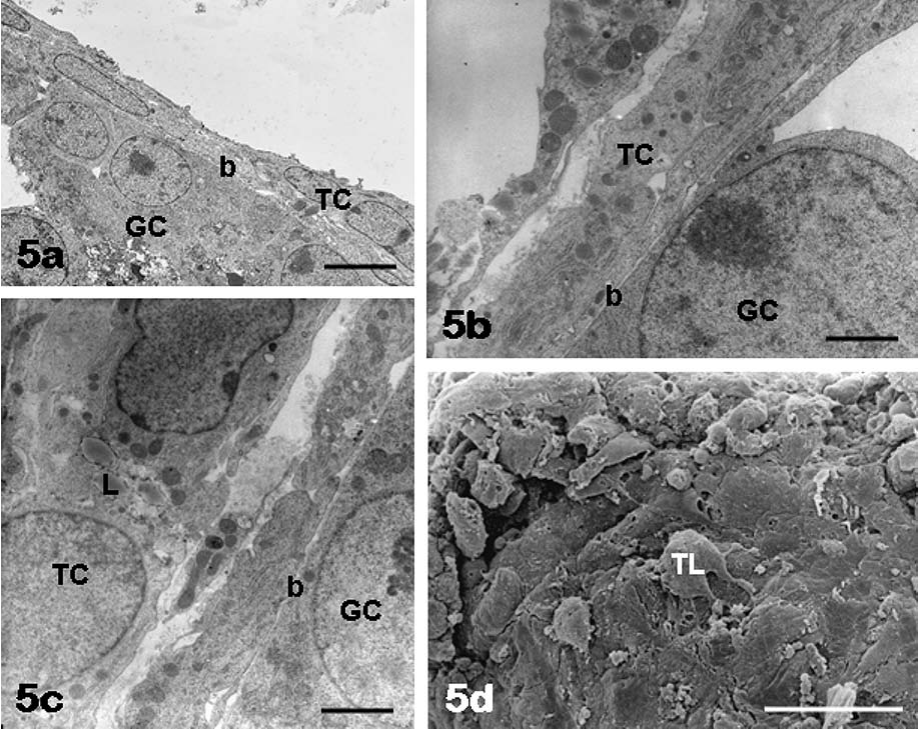
**FSH antral follicles: theca cells**. Theca cells (TC) forming one or two layers are seen by TEM in panels a, b. Lipid droplets (L) are visible in the theca cell cytoplasm (panel c). By SEM, the cells of the theca layer (TL) are provided with a few surface expansions (panel d). Panels a-c, GC: granulosa cells; b: basement membrane. Bar is: 5 μm (panel a); 0.9 μm (panel b); 1.5 μm (panel c); 30 μm (panel d).

*Granulosa layer: *the onset of antral formation caused loss of contact between GCs (arrows in Figure 4f). In 90% of the samples GC nucleus usually displayed dispersed chromatin, one or more nucleoli and a regular nuclear envelope (Figure 4f); their cytoplasm showed abundant mitochondria and lipid droplets (Figure 4g). Outer GCs appeared as irregularly rounded or polygonal cells (Figure 4h) arranged in numerous concentric layers, radially situated around the oocyte (Figure 1c, d) and delimited by a continuous basement membrane (Figure 5a, b, c). Only in a small percentage of follicles (10%) the GC population showed obvious ultrastructural features of cell damage.

*Theca layer: *in all samples TCs, arranged in one or two layers (Figure 1c, d; Figure 5a, b) showed a flat and elongated shape (Figure 5a, d), a large nucleus with numerous dense chromatin aggregates, a cytoplasm containing scarce lipid droplets (Figure 5a, b, c) and a few surface microvilli and blebs (Figure 5d).

#### FSH non-antral follicles

**Figure 6:**
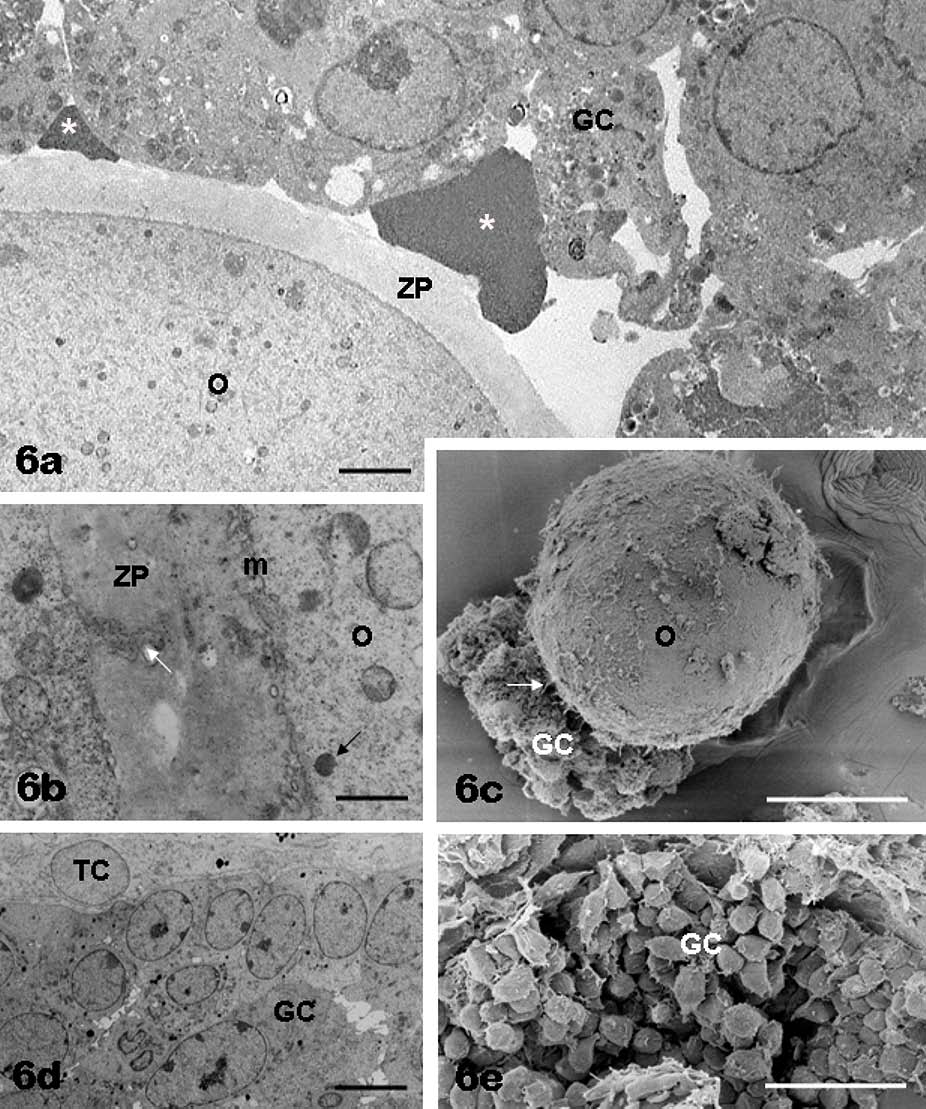
**FSH non-antral follicles: oocyte and granulosa cells**. In panel a the oocyte (O) contains numerous organelles scattered in the ooplasm. A variable amount of microvilli (m, panel b) can be found on the oocyte surface (O, panels b, c). Both altered (panel a) and apparently healthy (panel d) granulosa cells (GC), often provided with surface expansions (panels c, e), are seen in the follicle wall. Panel a, asterisks: granulosa cell debris. Panels a, b, ZP: zona pellucida. Panel b, black arrow: cortical granule. Panels b, c, white arrows: granulosa cell cytoplasmic projections (transzonal processes). Panel d, TC: theca cells. Bar is: 2.5 μm (panel a); 0.6 μm (panel b); 26 μm (panel c); 5 μm (panel d); 36 μm (panel e). Panels a, b, d: TEM; panels c, e: SEM.

*Oocyte: *the ooplasm showed scattered organelles (Figure 6a) and a few cortical granules placed in suboolemmal areas (Figure 6b). The oolemma was provided with a variable number of microvilli (Figure 6a, b, c). The zona pellucida was ultrastructurally normal (Figure 6a). TZPs of GCs were found crossing the zona pellucida and reaching the oocyte (Figure 6a, b, c); however, as far as the TZP number is concerned, it displayed an intermediate value in FSH non-antral follicles (mean number ± SD per 20 μm^2 ^= 14.20 ± 8.05) in comparison with the number of TZPs found in FCS and FSH antral follicles, and the difference was statistically significant in both cases (P = 0.0002 and P = 0.0334, respectively) (Table 1).

**Figure 7:**
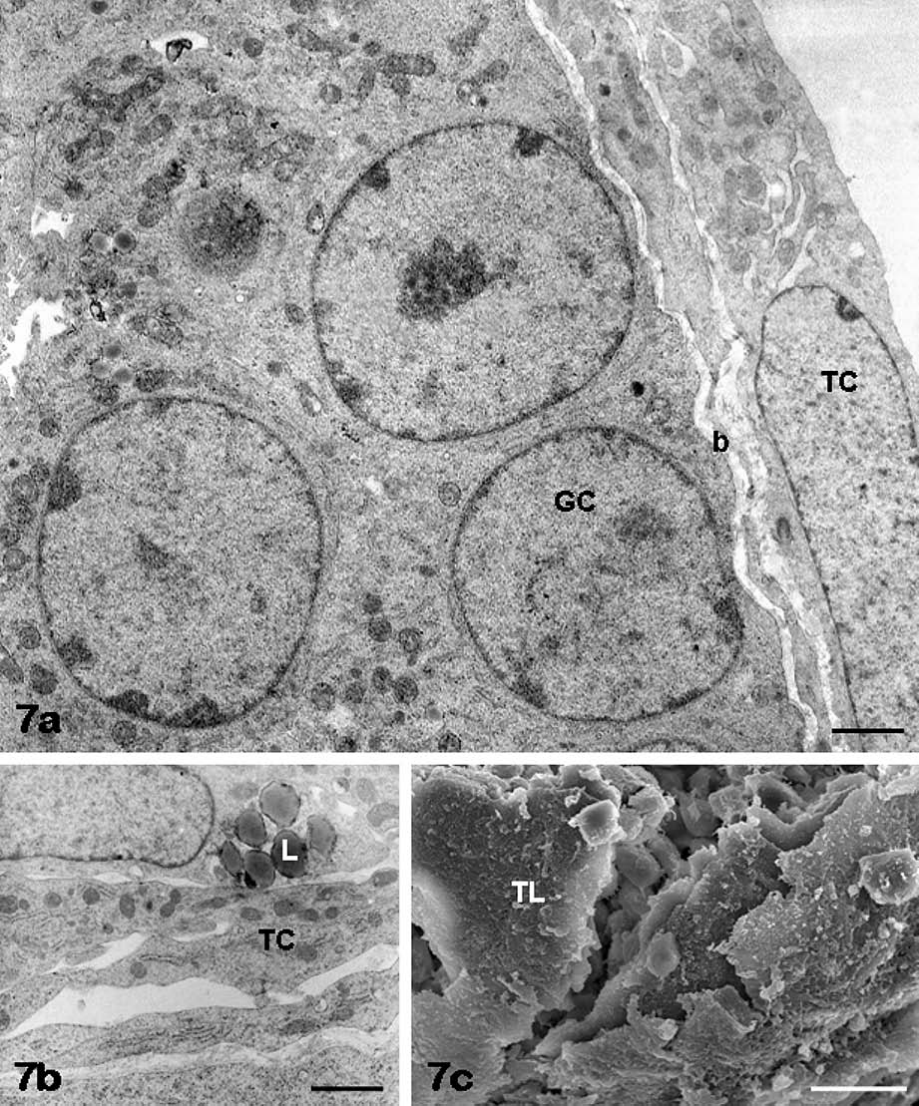
**FSH non-antral follicles: theca cells**. Theca cells (TC) forming one or two layers are seen by TEM in panels a, b. Lipid droplets (L) are visible in the theca cell cytoplasm (panel b). By SEM, the cells of the theca layer (TL) show a few surface expansions (panel c). Panel a, GC: granulosa cells; b: basement membrane. Bar is: 1 μm (panel a); 1.2 μm (panel b); 20 μm (panel c).

*Granulosa and theca layers: *overall ultrastructural morphology of FSH non-antral GCs (Figure 1e, f; Figure 6a, d, e) and TCs (Figure 1e, f; Figure 7a, b, c) was almost superimposable to that seen in FCS samples.

Table 2 shows a summary of Results.

**Table 2:** Ultrastructural changes in IVG mouse ovarian follicles cultured with or without FSH (100 mIU/ml) for 6 days

*Group*	*N°*	*Shape*							
			*Oocyte*			*Granulosa*			*Theca*
			*Organelle* *distribution*	*Oolemma*	*Zona* *Pellucida*	*N° and* *arrangement of* *layers*	* Transzonal* processes*	*Signs of* *degeneration*	*N° of* *layers*
***FCS***	20	Rounded	Juxta-nuclear	Microvilli +/-	Intact	4-5 Compact	++	Present in 40% of samples	1-2
***FSH*** ***ANTRAL***	20	Rounded	Scattered	Microvilli +	Intact	6-8 Cell loosening, with fluid-filled intercellular spaces Innermost layer discontinuous	+/-	Present in 10% of samples	1-2
***FSH*** ***NON-*** ***ANTRAL***	20	Rounded	Scattered	Microvilli +/-	Intact	6-8 Compact	+	Present in 40% of samples	1-2

*See Table 1 for details

## Discussion

It is well established that gonadotropins are necessary for antral follicle development *in vivo *[37-40]. Gonadotropins do not influence initiation of follicle growth, but they are critical to complete follicular development [1]. Even if preantral follicle growth *in vivo *is considered to be gonadotropin independent, FSH supplementation *in vitro *can be useful in driving initial follicle growth by exerting a positive effect on follicle survival and oocyte quality in mouse [23,60,61] and other mammalian species [31,34,62-65]. Some studies, however, report contrasting results [66,67]. FSH is also essential in inducing the production and/or effect(s) of factors that may positively affect follicle growth [68]. Also the modulator activity of activin on follicular culture [20,61,69] may be exerted through up-regulation of FSH receptor expression in GCs at early follicular stages [70].

Our study reports the morphological ultrastructural characterization of mouse preantral follicles cultured *in vitro *with intact theca layers according to the protocol established by Gosden and collaborators [24]. The results obtained demonstrated that IVG follicles forming AC present several ultrastructural features of maturity if compared with those grown *in vivo *[71-73]. Such a positive effect is mediated by the presence of FSH in the culture medium, as follicles cultured with FCS only were characterized by the failure of oocyte development and GC proliferation, and excess of GC degeneration as well.

Concerns from a data presentation perspective could arise from the obvious lack of a control, viable oocyte microscopic analysis from ovarian samples that would be physiologically comparable to the endocrine environment which defined these *in vitro *studies. Therefore, in order to support the interpretation of the noted structural changes in oocyte ultrastructure, we had to compare our *in vitro *results to the baseline *in vivo *data that were found in the literature. However, the changes noted between FCS (*in vitro *control) and FSH-treated follicles clearly demonstrated the supportive influences of FSH on oocyte viability *in vitro*.

Following FSH addiction to culture, the most evident characteristic of maturity shown by the FSH-treated group was represented by the rearrangement of oocyte intracytoplasmatic organelles, which appeared uniformly scattered throughout the cytoplasm. In FCS follicles, most of the organelles are instead condensed into a crescent region of the cytoplasm close to the nucleus, probably corresponding to the so-called "paranuclear complex". This feature is the ultrastructural correlate of the Balbiani's vitelline body and is typical of resting oocytes [47,74]. The persistence of this structure in oocytes of growing follicles may be a sign of ooplasmic immaturity [75] or may be associated with loss of oocyte viability, at least in the mouse [76]. Other ultrastructural markers of maturity in the oocytes of the FSH-treated group (and mostly in FSH antral follicles) were the distribution of cortical granules, that were seen throughout the ooplasm and in subplasmalemmal areas [47] and the numerous microvilli uniformly distributed on the plasmalemma. During follicular growth, the oocyte surface displays a gradual increase of microvilli, as clearly demonstrated by studies on macerated samples in rodents [44,45]. Microvilli seem to be involved in apposition and fusion of the sperm and oocyte membranes at fertilization [77,78]. Despite to the evidence of a cytoplasmic maturation in the oocytes of FSH-treated follicles, oocyte diameter does not increase up to the final size, reaching *in vitro *only 90% of the diameter of the oocytes physiologically grown *in vivo *[41,42,73].

By SEM and TEM analyses, in FSH-treated follicles, the GCs facing the oocyte appeared frequently detached, forming a discontinuous layer. This feature was particularly evident in FSH antral follicles. Long and tortuous TZPs crossing the zona pellucida and reaching the oolemma were present in the areas where GCs adhered to the oocyte. From a morphometric analysis the number of TZPs in FSH-treated follicles, both antral and non-antral, appeared reduced in comparison with that found in FCS follicles. This reduction was more evident in FSH antral follicles. In *in vivo *conditions, TZPs are very numerous in preantral follicles, forming both communicating (gap junctional) and adhesive contacts at the oolemma. The formation of gap junctions is fundamental for oocyte growth and maturation [46,79,80] because it facilitates the transfer of amino acids, glucose metabolites and nucleotides to the growing oocyte [46]. The TZPs could provide a polarized means that orients the secretory organelles of the somatic cells [81]. Presence of *zonulae adherentes *and desmosomes also guarantee the mechanical stability of the follicular unit [47,82]. During antral follicle development *in vivo*, TZPs retract and maintain fewer terminal connections with the oocyte than in preantral follicles. This consequently changes the oocyte transcriptional activity and meiotic competence. Such a retraction seems promoted by FSH, through a remodeling of the TZP cytoskeleton [83]. In our study, the reduction in number of TZPs in FSH-treated follicles, and particularly in those developing the AC (FSH antral follicles), well correlates with the data summarized above. However, in our *in vitro *model, such a reduction could be related not only to a physiological TZP retraction but also to the presence of areas of detachment, with consequent structural uncoupling, between the innermost GC layer and the oocyte.

The small percentage of morphological damages of GCs of FSH antral follicles can be understood as a physiological event occurring in follicular development in response to specific apoptotic signals or lack of survival signals [47]. The increased amount of degenerated figures such as changes of chromatin and blebbing of plasma membrane, found in both FCS and FSH non-antral follicles, are likely due to absence or partial inactivity of FSH, respectively [84]. In agreement with our ultrastructural data, the rate of apoptosis in the GC compartment is higher in FCS follicles than in FSH-treated follicles [42].

One or a few layers of TCs were found around the cultured follicles belonging to all groups. Theca layers may be damaged by follicle collection, remaining in a small number after mechanical isolation [50]. In all groups TCs presented an elongated and/or flattened shape with occasional cytoplasmic lipid droplets; these appearances are typical of immature TCs [47].

## Conclusions

We have evaluated a standardized culture system in which FSH is allowed to stimulate preantral follicle development *in vitro *until the antral stage. Our results provide the direct evidence that FSH addiction is essential for the morphological follicle differentiation during *in vitro *growth of mouse preantral follicles. Our study clearly demonstrates that only FSH-treated follicles show ultrastructural markers of maturity (AC formation, scattered organelles, retraction of TZPs). The different response to FSH and the delicate ultrastructural differences observed between FSH antral and FSH non-antral follicles suggest the existence of a process of commitment of certain follicles acting at the onset of development. Uncommitted follicles maintain granulosa compartment resistant to FSH and unable in forming AC. Other studies are required to confirm this hypothesis.

We also demonstrated that, even when FSH is administered and induces AC formation, not all the components of the cultured follicles undergo the morphological changes occurring *in vivo*. In fact, in all groups, including FSH antral follicles, oocytes grew but did not reach the full size, as the *in vivo *counterpart. The presence in FSH antral follicles of an abnormal, diffuse structural GC-oocyte uncoupling (beyond the physiological TZP retraction) may be the ultrastructural sign of an altered cross-talk between the gamete and the surrounding somatic cells. This may be a factor determining the inability of the oocyte to complete its growth *in vitro*. Thus, our study highlights the necessity of maintaining efficient intercellular communications to obtain *in vitro *large numbers of fully-grown mature germ cells.

Finally, FSH addiction has a limited effect on TC morphological maturation, which plays a crucial role in driving regular GC proliferation during preantral follicle development [85]. The absence of a fully mature theca layer around cultured follicles could be mainly related to the lack of blood vessels and stromal cells to be recruited at the periphery of the follicles. Consequently the follicles are isolated from systemic influences and are not exposed to vascular growth factors and to components of extracellular matrix. Perhaps a better development of TCs could be obtained by culturing the follicles within low-stiffness synthetic extracellular matrices [86] which mimic the *in vivo *microenvironment. Also GH addiction during culture could be useful in sustaining TC proliferation and differentiation [50].

In conclusion, several morphological discrepancies have been evidenced in our study between *in vivo *and *in vitro *development of mouse ovarian follicles, even when FSH was administered in culture to reduce the negative influence of the artificial microenvironment on ovarian follicle growth. Electron microscopy, associated with other *in vivo *and *in vitro *analytic studies, has a well recognized diagnostic-prognostic role in the assessment of ovarian follicle and oocyte viability during the application of biotechnological protocols in assisted reproduction, including those designed to fertility preservation [87,88]. The introduction of ultrastructural markers may be useful to evaluate the quality of IVG follicles, and particularly of their oocytes. Our original observations, as well, may ultimately serve as a model in research to improve knowledge in folliculogenesis and oogenesis, and may represent a basic reference for further morphologic studies on follicular somatic cells, oocyte and their interaction in *in vitro *models.

## Competing interests

The authors declare that they have no competing interests.

## Authors' contributions

SAN and SC directed the study and wrote the manuscript. SAN also participated to the interpretation of the ultrastructural data and performed the statistical evaluation. SC, SB, CM, GR, MAC have made contribution to acquisition of data. In particular, SC and GR provided the *in vitro *culture protocol, SB participated in the acquisition and evaluation of SEM data and helped to draft the manuscript, CM was involved in the acquisition of ultrastructural data and MAC participated in the acquisition and analysis of LM data. GM designed and directed the study, participated in the evaluation of data and critically revised the manuscript. Acquisition of funding was performed by SAN, SC and GM. All authors read and approved the final manuscript.
